# Dual Action of miR-125b As a Tumor Suppressor and OncomiR-22 Promotes Prostate Cancer Tumorigenesis

**DOI:** 10.1371/journal.pone.0142373

**Published:** 2015-11-06

**Authors:** William T. Budd, Sarah J. Seashols-Williams, Gene C. Clark, Danielle Weaver, Valerie Calvert, Emanuel Petricoin, Ema A. Dragoescu, Katherine O’Hanlon, Zendra E. Zehner

**Affiliations:** 1 Department of Biochemistry and Molecular Biology, Massey Cancer Center, Virginia Commonwealth University, Richmond, Virginia, United States of America; 2 Department of Forensic Science, Virginia Commonwealth University, Richmond, Virginia, United States of America; 3 Center for the Study of Biological Complexity, Virginia Commonwealth University, Richmond, Virginia, United States of America; 4 Center for Applied Proteomics and Molecular Medicine, George Mason University, Manassas, Virginia, United States of America; 5 Department of Pathology, VCU Medical Center, Virginia Commonwealth University, Richmond, Virginia, United States of America; 6 American International Biotechnology, Richmond, Virginia, United States of America; University of Kentucky College of Medicine, UNITED STATES

## Abstract

MicroRNAs (miRs) are a novel class of small RNA molecules, the dysregulation of which can contribute to cancer. A combinatorial approach was used to identify miRs that promote prostate cancer progression in a unique set of prostate cancer cell lines, which originate from the parental p69 cell line and extend to a highly tumorigenic/metastatic M12 subline. Together, these cell lines are thought to mimic prostate cancer progression *in vivo*. Previous network analysis and miR arrays suggested that the loss of hsa-miR-125b together with the overexpression of hsa-miR-22 could contribute to prostate tumorigenesis. The dysregulation of these two miRs was confirmed in human prostate tumor samples as compared to adjacent benign glandular epithelium collected through laser capture microdissection from radical prostatectomies. In fact, alterations in hsa-miR-125b expression appeared to be an early event in tumorigenesis. Reverse phase microarray proteomic analysis revealed ErbB2/3 and downstream members of the PI3K/AKT and MAPK/ERK pathways as well as PTEN to be protein targets differentially expressed in the M12 tumor cell compared to its parental p69 cell. Relevant luciferase+3’-UTR expression studies confirmed a direct interaction between hsa-miR-125b and ErbB2 and between hsa-miR-22 and PTEN. Restoration of hsa-miR-125b or inhibition of hsa-miR-22 expression via an antagomiR resulted in an alteration of M12 tumor cell behavior *in vitro*. Thus, the dual action of hsa-miR-125b as a tumor suppressor and hsa-miR-22 as an oncomiR contributed to prostate tumorigenesis by modulations in PI3K/AKT and MAPK/ERK signaling pathways, key pathways known to influence prostate cancer progression.

## Introduction

Transformation, growth and extravasation of cancer cells results from a variety of genetic and epigenetic modifications. In the past decade, there has been a growing body of literature reporting that aberrant expression of microRNAs (miRs) contributes to the development of several human cancers [1–8]. miRs are an important class of non-coding RNAs that affect post-transcriptional protein levels and, in the presence of external cues and environmental stressors, have the ability to induce rapid changes in the proteome allowing the cell to respond in a more precise and energy efficient manner [2–5]. Numerous cellular processes are affected by miRs, including differentiation, growth/ hypertrophy, cell cycle control and apoptosis [5,6].

Dysregulated miRs have been shown to contribute to oncogenesis by the loss of tumor suppressing miRs or increased expression of oncomiRs [1,9,10]. Either loss of tumor suppressors or increased expression of oncomiRs ultimately results in increased cell growth, proliferation, invasiveness or metastasis. Aberrant expression of even a single miR has the potential to influence a large number of cellular processes, as each miR can affect hundreds of downstream proteins, which preferentially regulate important key cell signaling nodes and transcription factors [11]. Networks analysis shows that targets of miRs have a significantly higher node degree than randomly chosen genes [11,12]. Perturbation of these highly interconnected molecules impacts cell health leading to disease.

Therefore, it seems necessary to integrate multiple sources of biological information to understand the perturbations underlying a given pathology from a systems level. High throughput technologies such as DNA microarray, genome sequencing, reverse phase proteomics, and qRT-PCR profiling allow for the simultaneous acquisition of thousands of data points. The intersection of multiple data sources increases the likelihood of identifying key pathways involved in cancer initiation, progression and metastasis. This work describes a unique combinatorial approach to understand the impact of miR dysregulation upon prostate cancer progression. Although miRs have been observed to be associated with prostate cancer, there is not a clear consensus on specific miRs that contribute to oncogenesis [7,8].

A novel isogenic prostate cancer cell line progression model was used as it is thought to closely mimic tumor progression as it occurs *in vivo* [13,14,15]. Integration of miR quantification profiles and proteomic data has the potential to increase the success of identifying novel miRs affecting tumorigenesis. Therefore, the data generated from miR arrays, Reverse Phase Microarray (RPPA) proteomics, along with information from a previous networks analysis was used to rank dysregulated miRs in our prostate cancer progression model [12,16–18]. Putative dysregulated miRs were further verified in RNA isolated from patients’ radical prostatectomy tumor samples using laser capture microdissection [LCM] [19,20]. Several dysregulated miRs that potentially drive prostate cancer progression were identified [18,21]. Of these miRs, the importance of hsa-miR-125b (miR-125b) and hsa-miR-22 (miR-22) to tumorigenesis was further confirmed by *in vitro* experiments. miR-125b and miR-22 may be useful as relevant biomarkers to identify and stage prostate cancer.

## Materials and Methods

### Derivation of Prostate Cell Lines and Cell Culture

p69, M2182, and M12 cells were a gift from Dr. Joy Ware, Virginia Commonwealth University, Richmond VA, and authenticated using STR analysis [13,14]. These cell lines were derived by immortalization of a non-neoplastic prostate epithelium with SV40 large T antigen [13]. The parental (P69) cell line is poorly tumorigenic, and non-metastatic with a lower modal chromosome number than most other prostate cancer cell lines typically isolated from metastatic sites (LNCap, DU145, and PC3). An *in vivo* selection process was used to create cells with increased tumorigenicity and metastatic potential. After three rounds of subcutaneous injections into male athymic nude mice, a highly tumorigenic and metastatic variant (M12) was isolated, which routinely metastasizes upon intra-prostatic injection [14]. The M2182 cell line was derived after two rounds of *in vivo* selection and therefore represents an intermediate phenotype that is less tumorigenic than the M12 cells and is non-metastatic. Cells were kept in culture at 37°C for less than two months in RPMI1640 media with L-glutamine (Gibco), supplemented with 5% fetal bovine serum, 0.05 mg/ml gentamycin, 5μg/ml insulin, 5 μg/ml transferrin, and 5 μg/ml of selenium (ITS from Collaborative Research Bedford, MA) [13,14,19]. M12 cells stably transformed with a pSIREN plasmid vector (Clontech) expressing miR-125b (M12+miR-125b) were maintained with puromycin (100 ng/ml) [19,22]. M12 cells stably transformed with a miARREST^™^ miR22-3p inhibitor (M12+miR-22i) expression plasmid (pEZX-AM03) from GeneCopoeia (HmiR-AN0332-AM03) were maintained with hygromycin (200 μg/ml). Following trypsinization (0.25% in EDTA), cells were pelleted, washed with PBS, flash frozen in liquid nitrogen, and stored at -80°C until used.

### Cell Pellet RNA Extraction

Total RNA was extracted from frozen cell pellets using the miRVana^™^ miR isolation method (Ambion-Life Technologies) per manufacturer’s instructions. After isolation, RNA concentration was estimated using a Biorad^®^ Smart Spec^™^3000 spectrophotometer, diluted to a concentration of 100 ng/ml and stored at -80°C.

### Cell Pellet DNA Extraction

DNA was extracted from frozen cell pellets using the QIAamp DNA mini kit from Qiagen (Cat. No. 51304) following manufacturer’s instructions. Following extraction DNA concentration was measured as above.

### Onco Library and Sequencing method

DNA libraries were generated from the cell lines using the Ion AmpliSeq^™^ Library Kit 2.0 and the Ion AmpliSeq^™^ Cancer Hotspot Panel v2 (Cat. No. 4480441 and 4475346). The Cancer Hotspot Panel is a single amplicon pool that amplifies 207 amplicons covering over 2,800 COSMIC mutations from 50 known oncogenes and tumor suppressor genes. Each sample was prepared using 10 ng of input DNA according to the Ion AmpliSeq^™^ Library Preparation protocol (Publication Part Number MAN0006735 Revision A.0). Ion Xpress^™^ Barcode Adapters 1–16 Kit (Cat. No. 4471250) were utilized for individual sample barcoding and adaptor ligation. Agencourt AMPure XP magnetic beads (Cat. No. A63882, Beckman) were utilized as indicated in the Ion AmpliSeq^™^ Library Preparation User Guide. Sample libraries were quantitated using the Qubit 2.0 Fluorometer and the High Sensitivity dsDNA Assay Kit (Cat. No. Q32851) and normalized to a concentration of 100 pM. Emulsion PCR was performed on the Ion OneTouch^™^ 2 Instrument (Cat. No. 4474778) as indicated in Ion PGM^™^ Template OT2 200 Kit (Publication Number MAN0007220. Revision 5.0) using the Ion PGM^™^ Template OT2 200 Kit (Cat. No. 4480974). Normalized 100 pM sample libraries were pooled and combined with OT2 kit reagents and Ion Sphere Particles (ISPs). The reaction was run on the OT2 using the 200 bp protocol. Samples were then processed for enrichment on the Ion OneTouch^™^ ES. Enrichment of ISPs was achieved using the Ion PGM^™^ Template OT2 200 Kit (Cat. No. 4480974) and DynaBeads MyOne streptavidin C1 beads (Cat. No. 65001 Life Technologies) according to the manufacturer's protocols (Ion PGM^™^ Template OT2 200 Kit User Guide). The streptavidin C1 beads bind specifically to enriched ISPs, allowing all others to be washed away, followed by elution with a sodium hydroxide and tween melt solution. The Ion Torrent Personal Genome Machine (PGM) was initialized using the Ion PGM^™^ Sequencing 200 Kit v2 according to manufacturer’s recommendations (Ion PGM^™^ Sequencing 200 Kit v2: Publication Number MAN0007273 Revision 3.0). Control beads were spiked into the enriched bead sample, sequencing primers were annealed to the ISPs and polymerase added. ISPs were sequenced utilizing an Ion Torrent Ion 318^™^ Chip. The Ion Torrent PGM was run according to Ion Torrent 200 Kit v2 specifications utilizing 500 nucleotide flows, followed by alignment to the HG19 reference and analyzed by the Ion Torrent Variant Caller plugin.

### TaqMan^®^ based miR assay

Verification of mature miR expression was confirmed using TaqMan^®^ miR methodology (Life Technologies, Grand Island, NY). cDNA was synthesized in a 25 μl reaction volume from total RNA (20 ng) using the TaqMan^®^ MicroRNA Reverse Transcription kit. Reactions were incubated at 16°C for 30 min, 42°C for 30 min, and inactivated at 85°C for 5 min. Each cDNA was analyzed in triplicate by quantitative PCR (qRT-PCR) using the sequence specific primers hsa-miR-125b-1 (ID 000449), hsa-miR-22-3p (ID 000398) or RNU48 (ID 001006) in an Applied Biosystems 7300 real-time PCR instrument (Life Technologies). Each individual assay was performed in a 20 μl reaction volume with 1.33 μl of cDNA, 1.0 μl specific miR assay, 10 μl TaqMan^™^ Universal PCR Master Mix II with no AmpErase UNG and 7.67 μl of nuclease free water. Reactions were incubated at 50°C for 2 min, followed by 10 min at 95°C and 40 cycles with denaturation for 15 sec at 98°C, and annealing/extension for 60 sec at 60°C. Data was analyzed using SDS software v1.3.1 (Life Technologies). Threshold and baseline settings were set according to protocol recommendations, with baseline correction between cycles 3 and 12 and an automatic threshold.

### Statistical Analysis of qRT-PCR Data

Relative quantities of miR levels were determined using the 2^-ΔΔCT^ method of Livak et al after normalization with RNU48 as a standard reference [23]. All samples were performed in triplicate and the average cycle threshold (C_T_) value was determined from C_T_ values in the range of 25 to 35. The average C_T_ was normalized by subtracting the average C_T_ of RNU48 (ΔC_T_). All samples were compared to the parental P69 cell line by subtracting the experimental ΔC_T_ from the ΔC_T_ value of the p69 cell line as the calibrator. Data are presented as the mean ± standard deviation of 3 independent experiments. Statistical analyses were conducted in the Microsoft^®^ Excel software platform, using an independent Student's t-test, assuming equal variance between the two groups. Statistical significance was defined as p≦0.05.

### Laser Capture Microdissection (LCM)

Tumor and benign prostate tissues were obtained from radical prostatectomy samples, after approval from Virginia Commonwealth University (VCU), Office of Research and Innovation, Institutional Review Board (IRB), Panel A. Four samples were frozen tissue from VCU’s Tissue and Data Acquisition and Analysis Core (TDAAC at http://www.pathology.vcu.edu/research/TDAAC) and one archived FFPE sample was from VCU’s Department of Pathology [19,22]. Human tissues, patient consents, and clinical data were provided by the VCU TDAAC core facility and the Department of Pathology with patient consents. All patient samples were de-identified to protect patient confidentiality. Slides (10–20) were generated and reviewed by a board certified pathologist (ED) with expertise in prostate cancer diagnosis to identify prostatic adenocarcinoma foci versus normal glandular epithelium as well as hyperplastic glandular epithelium (BPH), prostatic intraepithelial neoplasia (PIN) and stroma from one unique patient sample. Prostatic adenocarcinoma was graded according to the Gleason grading system [24]. Briefly, 8 μm tissue slices were placed on uncharged glass slides, dehydrated with progressively increasing concentrations of ethanol, and stained with hematoxylin and eosin (H&E) [19,20,25–29]. LCM was performed using an Arcturus Veritas^™^ laser capture microdissection system (Life Technologies). Areas of interest (benign versus tumor plus stroma, BPH and PIN from one FFPE sample) were individually captured onto CapSure^®^ Macro LCM caps (Life Technologies, Grand Island, NY).

### RNA Extraction of LCM Samples

Total RNA was extracted from LCM captured material using the Arcturus^®^ PicoPure^®^ RNA Isolation Kit (Life Technologies, Grand Island, NY) according to the manufacturer’s protocol. Following tissue dissection, the LCM caps were incubated for 30 min at 42°C in 50 μl of extraction buffer and stored at -20°C until ready for RNA extraction. Cellular lysates from successive slides were pooled into a single tube and mixed with 1 volume of ethanol (70%). The lysate/ethanol solution was loaded onto a high recovery MiraCol^™^ column and centrifuged at 1000 xg for 2 min followed by 16,000 xg for 30 sec. RNA was recovered in an 11 μl volume. RNA concentration and quality were measured using an Agilent RNA 6000 Pico chip with the Bioanalyzer 2100 (Agilent Technologies) using the manufacturer’s instructions. All samples used for qRT-PCR displayed RNA of good quality as assessed by its RNA Integrity Number (RIN) and of sufficient quantity to not require a pre amplification step for subsequent PCR analysis. RNA (40 ng) was reverse transcribed for miR analysis using the qRT-PCR conditions described above.

### Luciferase+3’-UTR Reporter Assays

The 3’-UTR of the human ErbB2 gene (from -1 to -576 downstream of the stop codon) and referred to as pErbB2wtUTR was kindly provided by Dr. Christopher C. Benz [30]. The entire 3’UTR (-1 to -576) as well as subregions from -44 to -576 or -110 to -576 were retrieved by PCR using the appropriate 3’-UTR primers and the pErbB2wtUTR plasmid as template and cloned into the Xba site of the pmirGLO dual luciferase expression vector (Promega) to generate pErbB1/576, pErbB44/576 and pErbB110/576, respectively. The 3’-UTR sequence of the wild type and deleted subclones was verified by DNA sequencing. M12 cells (200,000 cells per well in a 6 well dish plated the day before) were transfected in triplicate with 1 μg of pmirGLO empty vector or plasmid containing the individual 3'-UTR regions from pErbB2 as described above. Transfection was accomplished using the TransIT^®^-LT1 Transfection Reagent (Mirus BIO LLC) according to manufacturer’s recommendations. A mix of reagent (2 μl TransIT^®^-LT1 /ng DNA), plasmids, and serum free RPMI media were incubated for 30 min at room temperature prior to addition to cells and incubated under normal cell culture conditions for 48 hours followed by collection and lysate preparation according to manufacturer’s instructions. Luciferase and renilla activity were measured in 30 μl of cell lysate using the Dual Luciferase Assay System (Promega Corporation) and a GloMax^®^ 20/20 luminometer (Promega Corporation). Reporter activity is expressed as the ratio of firefly to renilla activity. Each assay was repeated three times and the standard deviation within each sample is noted.

A miR-22 binding site within PTEN was identified by Bar et al.[31]. A top strand (56 bases of sequence 5’-CATATTGG***TGCTAGAAAAGGCAGCTAAAG****GAAGTGAATCTGTATTGGGGTACAGGT*) and bottom strand (62 bases of sequence ***5’- (*** ***TCGAGTATAACCACGATCTTTTCCGTCGATTTCCTTCACTTAGACATAACCCCATGTCCAGC*** 62 bases) with the centrally located miR-22 target region (***in italics***) was synthesized and directionally cloned into the Sac/AccI site of pmirGLO (Promega). A mutant PTEN 3’-UTR sequence containing a C (**red)** to A mutation which has been shown to abolish regulation by miR-22 binding was similarly synthesized and cloned [31]. Both wild type and mutant PTEN 3’-UTR sequences were confirmed by DNA sequencing. Transient transfections into the M12 cell line were completed as described above except 250 ng of the various pmirGLO plasmids was found optimal for transfection.

### Western Immunoblotting

Whole cell extracts were prepared as previously described [22]. Protein (30 μg) was analyzed on a Novex^®^ 4–12% Tris-Bis gradient polyacrylamide gel and immunoblot analysis performed as described previously [32]. Primary antibodies used were β-actin (1:200) produced in mouse (C4:Santa Cruz Biotechnologies Inc.) and PTEN (1:1000) produced in rabbit (D4.3:Cell Signaling). After incubation in primary antibody overnight, the blot was washed as previously described [32] and incubated first in secondary anti-mouse IgG-HRP (1:1000) produced in goat (Santa Cruz Biotechnologies) and then with anti-rabbit IgG-HRP (1:1000) produced in goat (Cell Signaling) for 1 hour at room temperature. After washing bands were developed using the chemiluminescent reagents from Western Lightning^®^ (Perkin Elmer, Boston, MA). Western blots were exposed and bands quantitated using the ODYSSEY^®^ Fc Imaging System (LI-COR Biosciences, Lincoln, NE).

### Cell Proliferation Assay

The proliferation of M12 versus the stable transformed cell lines, M12+miR125b and M12+miR-22i was compared. Cells were trypsinized, washed free of trypsin with serum-containing media, and plated in triplicate (20,000 cells/well) in 9-well plates. The viable cell number was quantitated at 0, 48, and 72 hours with a Beckman Coulter Vi-CELL-XR Automated Cell Viability Analyzer using a trypan blue solution. The average viable cell number was plotted on an exponential curve with the standard deviation noted. Cell growth was compared at 0, 48 and 72 hour time points using a student t-test yielding a p-value of 0.11 and 0.009, respectively.

### Migration Assay

Migration and invasion assays were performed as previously described [19,22]. Cells were detached from the plate using 2.0 ml of CellStripper^™^ (CellGro^®^, Manassas, VA), pelleted, washed in serum free RPMI 1640 and re-suspended at a concentration of 2.5 x 10^5^ cells/ml. A cell suspension (50,000 cells in 200 μl) was added to the top chamber of a 6.0 μm pore size ThinCert^™^ tissue culture insert (Greiner Bio-one BVBA/SPRL, Monroe, NC). Serum containing media (O.5 ml) supplemented with 10 ng/ml of EGF was added to the lower chamber. Cells were incubated at 37°C for 20 hours. Media was removed from both chambers and cells that did not invade (top surface) were removed with a sterile cotton applicator. Cells on the lower surface were fixed in 0.025% glutaraldehyde in PBS for 20 min. Following fixation, inserts were stained with 0.1% leucocrystal violet in 10% ethanol and PBS for 30 min and washed with sterile deionized water. Membranes were excised and mounted on a glass microscope slide. Cells were counted in 10 random fields at a 200 X magnification. Data is presented as the mean sum of migratory cells in 10 random fields ± standard error.

### Invasion Assay

Invasion assays were carried out in a manner similar to the migration assays. At least 30 min prior to extracting cells, 60 μl (diluted 1:10) of Culturex^®^ reduced growth factor basement membrane (Growth Factor Reduced) was added to the top chamber of the ThinCert^™^ tissue culture insert and incubated at 37°C for gelling. The remainder of the protocol is identical to the migration assay described above. Data is presented as the mean sum of invasive cells in 10 random fields ± standard error.

### Reverse Phase Microarray (RPPA)

RPPA experiments were conducted at the George Mason University Center for Applied Proteomics and Molecular Medicine under the supervision of Dr. Emanuel Petricoin III [16,17]. To minimize variation duplicate cell pellets were used for RNA extraction for miR profiling and for proteomics [18]. For RPPA, cell pellets were harvested by scrapping, washed with PBS, flash frozen and stored at -80°C until used. Pellets were directly lysed in a tissue extraction buffer and spotted on a nitrocellulose coated glass slide (Grace Bio-labs, Bend OR) in a miniature dilution curve (1:1, 1:2, 1:4, 1:8, and 1:16) using an Aushon 2470 arrayer (Aushon BioSystems Billerica, MA) to ensure accurate quantification of each protein measured [16]. Three successive passages for each cell line were analysed with each sample printed in triplicate along with standard curves for internal quality control. Selected arrays were stained with Sypro Ruby Protein Blot Stain (Molecular Probes, Eugene, OR) following manufacturing instructions to quantify the amount of protein present in each sample. Prior to antibody staining the remaining arrays were treated with Reblot Antibody Stripping solution (Chemicon, Temecula, CA) for 15 min at room temperature, washed with PBS, and incubated for at least one hour in I-block (Tropix, Bedford, MA). Using an automated system (Dako Cytomation, Carpinteria, CA), arrays were first probed with a 3% hydrogen peroxide, biotin blocking system, and then an additional serum free protein block to reduce nonspecific binding between endogenous proteins and the detection system. Antibodies were validated for their use on the array by verifying single-band Western blotting and peptide completion/ligand induction. ErbB2/3, pBAD, pERK and MET were rabbit monoclonal antibodies, PI3K a rabbit polyclonal, all supplied by Cell Signaling, Inc. Biotinylated anti-rabbit (Vector Laboratories, Inc. Burlingame, CA) or anti-mouse secondary antibody (CSA; Dako Cytomation Carpinteria, CA) coupled with the Catalyzed Signal Amplification System (CSA; Dako Cytomation Carpinteria, CA), a commercially available tyramide-based avidin/biotin amplification kit, were employed to amplify the detection of the signal. Fluorescent detection was obtained using IRDye 680RD Streptavidin (LI-COR Biosciences, Lincoln, NE) according to the manufacturer’s recommendation. Antibody and Sypro Ruby stained slides were scanned on a Tecan laser scanner (TECAN, Mönnedorf, Switzerland) using the 620 nm and 580 nm weight length channel respectively. Images were analysed with MicroVigene Software Version 5.1.0.0 (Vigenetech, Carlisle, MA) as previously described [16]. Overall, the cellular lysate was spotted onto 111 slides and each incubated with a unique antibody. Data was returned on a Microsoft Excel spreadsheet containing each cell type analyzed with each antibody of interest.

### Statistical Analysis of the RPPA Data

Microsoft Excel was used to perform a two-sample equal variance T-test to compare the protein expression data between the M12 and p69 cell lines. A total of 45 values was averaged for each antibody probed protein sample. A strict probability value of p < = 0.001 was used to correct for variation among multiple samples and to identify truly significant changes. The reported significant changes were converted into fold changes by dividing the average expression of the protein in the M12 cell line by the average expression of the protein in the comparative p69 cell line.

## Results

### Next Generation Sequencing of Prostate Cancer Cell Lines

The p69, M2182, and M12 cell lines represent an unique set of genetically related prostate cancer cell lines proposed as a progression model duplicating the molecular events that occur during prostate tumorgenesis *in vivo* [13,14,15]. The M12 cell line is a highly tumorigenic/metastatic variant derived from the parental p69 cell line with the M2182 cell line displaying an intermediate, but non—metastatic phenotype. In contrast, other established human prostate cell lines such as Du145 or PC3 represent cellular end points derived from secondary tumors and thus, cannot be viewed as a progression model.

Next generation sequencing (NGS) analysis was undertaken to identify potential nucleotide variations in genes known to be associated with cancer progression that could account for the phenotypic differences displayed by these cell lines. Over 200,000 reads were obtained for each subline with a mean read length of ~110 nts in length (Table 1). The Catalogue of Somatic Mutations in Cancer (COSMIC) describes over two million coding sequence point mutations identified in various cancers. However, there were only three COSMIC gene mutations identified in all sublines (STK11-COSM29005, PGFRA- COSM22413 and APC-COSM19099) (Table 2). Interestingly, all of the COSMIC mutations plus an additional 11 novel mutations were consistent across all members (P69, M2182 and M12) of the prostate cancer cell progression model. There was a single transition from a thymidine to a cytosine in the STK11* gene, which was not present in the M12 cell line, but since this is NOT a mutation known to correlate to cancer risk, it is unlikely that it is solely responsible for the unique properties of this subline. Thus, the NGS analysis showed that there were no previously known cancer gene mutations that could account for the vast differences in tumorigenicity and metastasis displayed by these related cell lines.

**Table 1:** Next generation sequencing results in prostate cancer cell line model.

Cell Line	Reads	Mean Length	Total Bases
P69	562,713	110	61,626,082
M2182	206,177	114	23,518,988
M12	448,016	114	50,956,206

**Table 2:** Single nucleotide variations in prostate cancer cell line model.

Gene Symbol	Chromosome	Position	Reference Nucleotide	Alternate Nucleotide	Cosmic ID
PIK3CA	chr3	178917005	A	G	—
MET	chr7	116340269	C	T	—
NOTCH1	chr9	139390853	C	T	—
RET	chr10	43613843	G	T	—
TP53	chr17	7579472	G	C	—
STK11*	chr19	1220321	T	C	—
STK11	chr19	1221293	C	T	COSM29005
PIK3CA	chr3	178927410	A	G	—
FGFR3	chr4	1807894	G	A	—
PDGFRA	chr4	55141055	A	G	—
PDGFRA	chr4	55152040	C	T	COSM22413
KDR	chr4	55955139	A	T	—
APC	chr5	112175240	G	C	COSM19099
APC	chr5	112175770	G	A	—
CSF1R	chr5	149433596	TG	GA	—

*Not present in M12 cell line

### Analysis of miR Expression in Prostate Cancer Cell Lines

**Fig 1:**
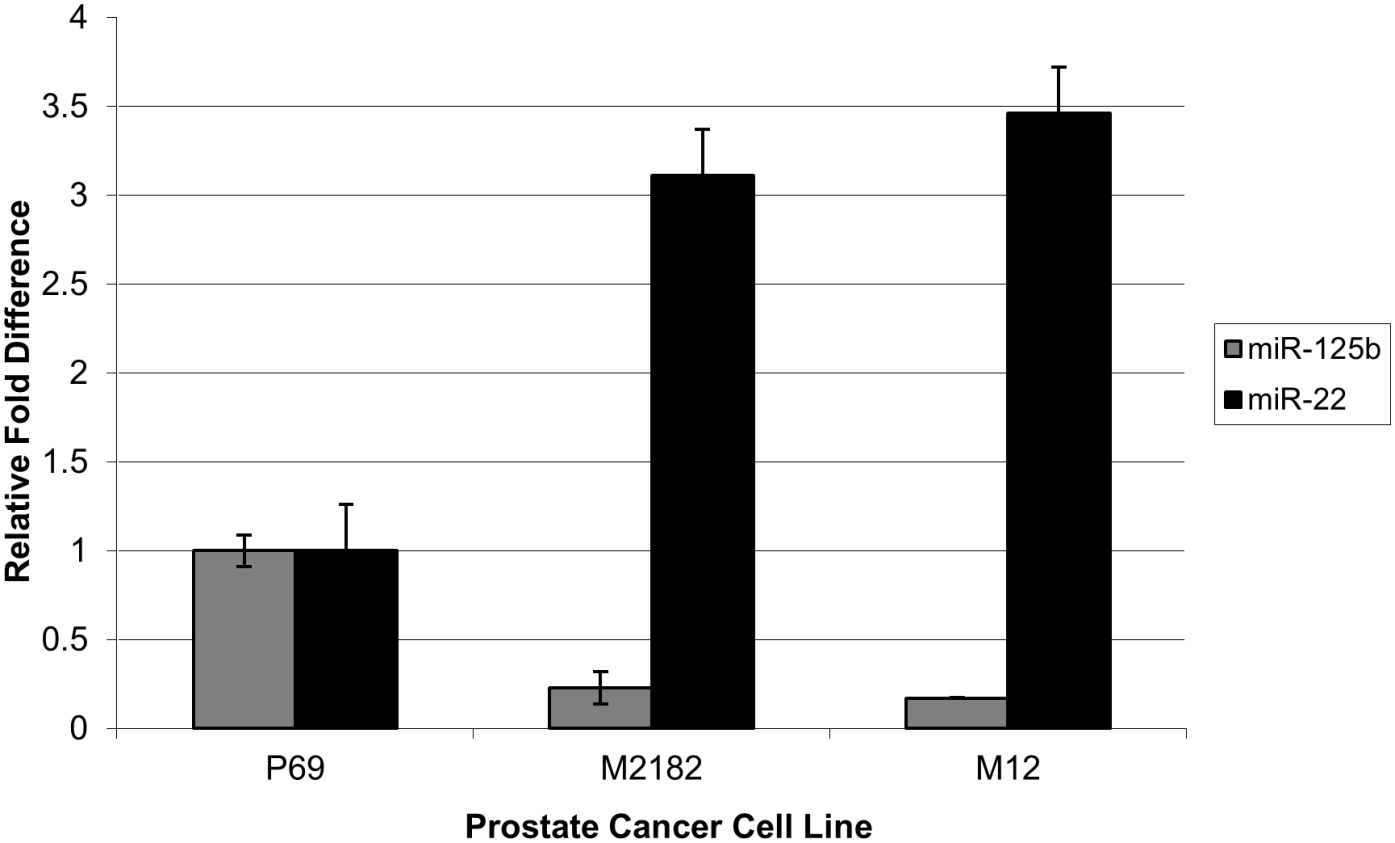
Dysregulation of miR-125b and miR-22 in the p69/M12 human prostate cancer cell line progression model. Comparison of miR-125b (grey) and miR-22 (black) levels in RNA extracted from p69, M2182 and M12 cell lines by SYBR based qRT-PCR as described in Materials and Methods. Fold difference was calculated as the mean of three independent experiments analyzed in triplicate and normalized to RNU48 (ΔC_T_) as an internal control and expressed relative to the parental p69 cell line using the comparative C_T_ method [23]. ANOVA test indicates a significant difference with a P-value < 0.05.

To identify genes that could contribute to the different tumorigenic/metastatic properties of these cells, a miR array screen using the miRCURY LNA ^™^ Universal RT microRNA PCR system (Exiqon, Denmark) was conducted in duplicate [18,21]. Although several miRs were found to be dysregulated, most notable was the differential expression of miR-125b and miR-22 [18,21]. As the prostate cell lines became more tumorigenic progressing from the parental p69 to the M2182 and ultimately the M12 cell, the level of miR-125b decreased substantially, ≈ a 6-fold drop in expression (Fig 1). In contrast, miR-22 likely functioned as an oncomiR with the level of miR-22 increasing 3.5-fold as the cells became more tumorigenic and ultimately metastatic. Interestingly, in both cases the bulk increase occurred during the early phases of tumorigenesis from P69 to M2182 with only slight additional changes in the transition from M2182 to the M12 cells.

### MiR Expression in Tumor Tissue RNA Extracted by LCM

**Fig 2:**
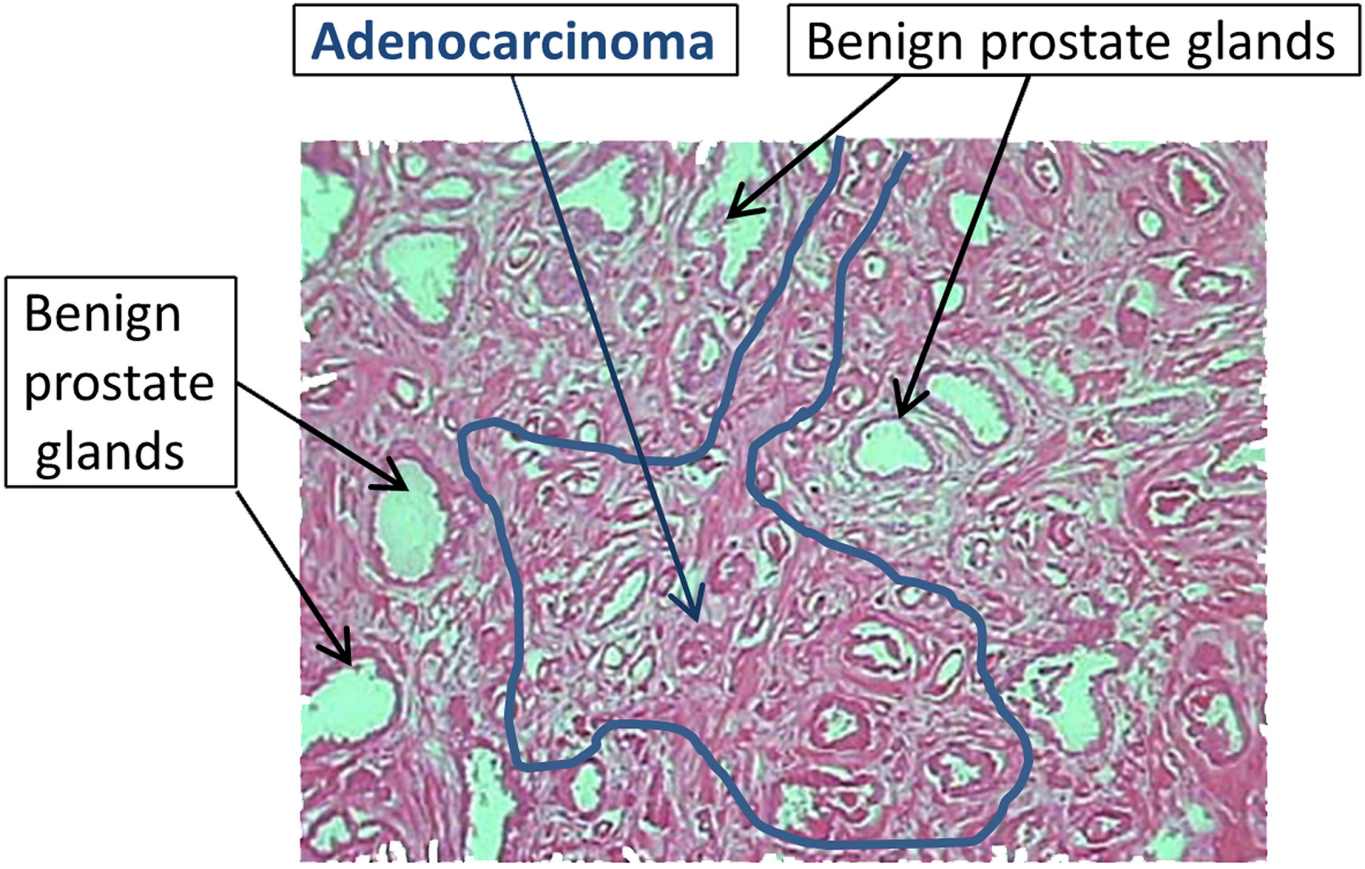
LCM is required to minimize variation due to tissue heterogeneity of human prostate tissue. This image of a frozen human prostate sample exemplifies the heterogeneity typical of human tissues: areas of adenocarcinoma, outlined in blue, are intermixed with stroma and benign prostate glands, labeled by arrows. LCM allows for targeted dissection of the malignant glands (scored as G3 and G4 for this particular sample) while avoiding the stroma and other benign structures.

**Fig 3:**
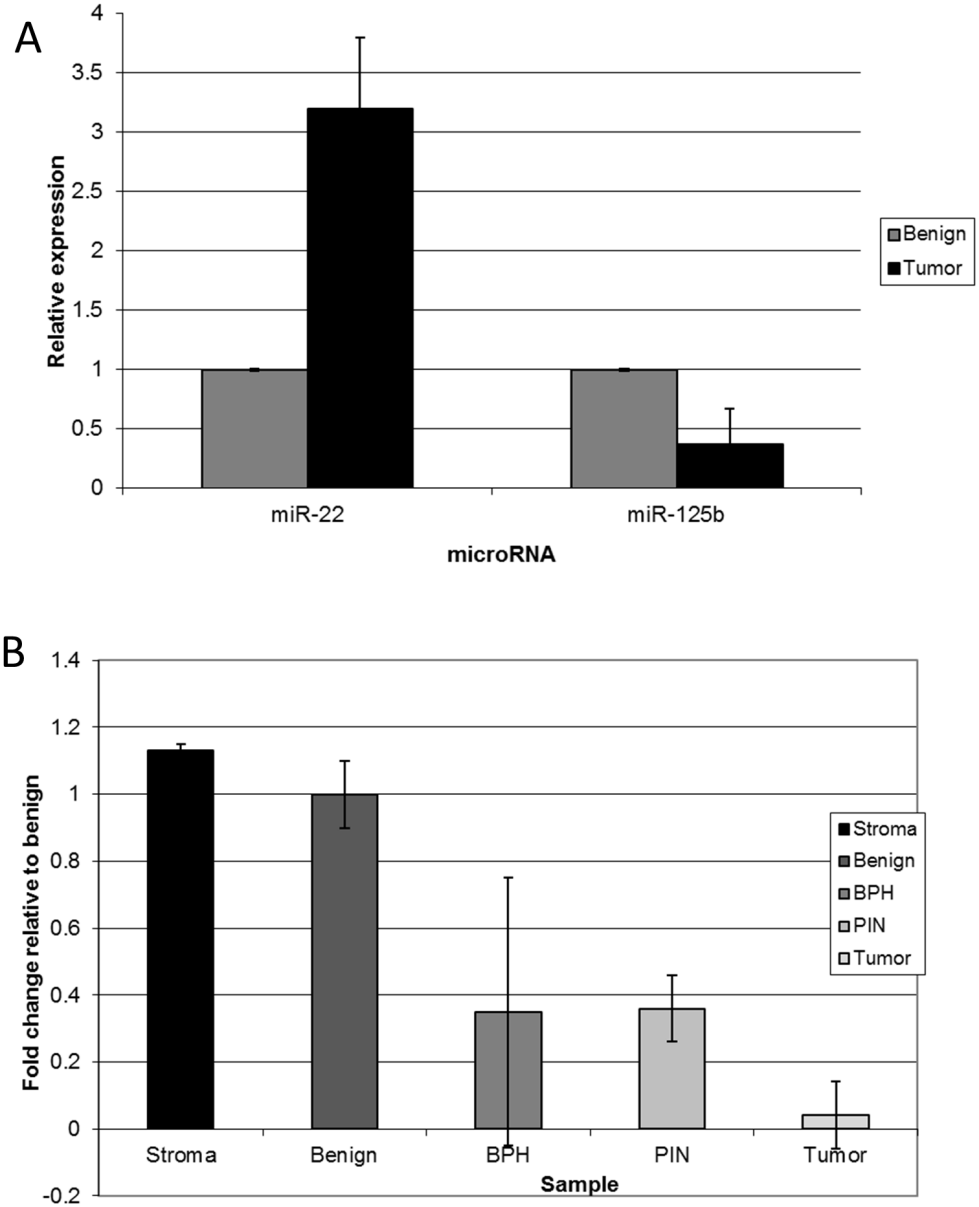
Dysregulation of miR-125b and miR-22 expression in RNA extracted from prostate tissue samples by LCM. **(A)** Frozen prostate samples were dissected via LCM into benign glandular epithelial versus tumor. Total RNA was extracted using the PicoPure RNA extraction method, as described in Materials and Methods. qRT-PCR was performed with specific primers against miR-125b and miR-22 using the TaqMan^®^ Universal Master Mix II. C_T_ values were normalized to RNU48 and reported as fold differences compared to benign tissue using the ΔΔCT method of Livak et al. [23]. Results are the average from frozen tissue samples (n = 4) repeated in 3 technical replicates. **(B)** From 10 successive slides of a single FFPE prostate sample, miR-125b was quantitated by qRT-PCR as described in panel A for RNA extracted from stroma, benign glandular tissue, BPH, PIN and tumor of stage G4.

To confirm those findings observed with prostate cancer cell lines, miR dysregulation was assessed in human tumors obtained after radical prostatectomy. Determination of molecular changes contributing to pathogenesis in the prostate is complicated by the heterogeneity of the tissue. Frequently, tumor of varying Gleason scores are surrounded by normal glandular tissue all embedded in stroma as noted (Fig 2). Pure cell populations can only be obtained from such a heterogeneous tissue with techniques like LCM [19,20,25–29], which allows for the direct visualization and collection of selected cells, yielding a pure source of RNA for downstream analyses. For example, an adjacent region from the tissue sample illustrated in Fig 2 contained prostatic urethra, and if it had been included, as it would have been in the analysis of a whole tumor sample, the results could have been significantly compromised. Thus, normal glandular epithelium or selected tumor cells captured with the exclusion of stroma or other benign elements are the best source for subsequent miR analysis. Comparing matched tumorigenic epithelium to its benign counterpart, we confirmed the findings of our *in vitro* cell line model. The level of miR-125b was significantly lowered (2.5-fold) in tumor cells compared to benign epithelium where miR-22 was ≈3.2-fold higher (Fig 3A).

In contrast with frozen tissue, dissection of FFPE samples is not time limited and provides an ideal opportunity to more finely resolve changes that occur during tumor progression by the collection of graded tumor RNA free from benign epithelium or stroma. In contrast, the major disadvantage of using FFPE samples is the limited quantity of extracted RNA as a higher quantity was consistently obtained from frozen samples (Fig 3A). For this reason, only a single miR could be chosen for further evaluation in an interesting FFPE sample. This patient’s sample displayed a wider range of cell- and tumor-types that could be used to further document the pattern of miR-125b activity during the tumorigenic process (Fig 3B). Benign prostatic epithelium, stroma, benign prostatic hyperplasia (BPH), prostatic intraepithelial neoplasia (PIN), and tumor were separately dissected by LCM and analyzed. BPH is a common disorder affecting nearly all men at some point in their lifetime and in general it is not thought to increase a man’s risk of developing prostate cancer [33]. The level of miR-125b decreased in BPH cells when compared to normal benign epithelium (Fig 3B). However, there was a great range of miR-125b levels in BPH cells as evident by the large standard deviation. PIN is considered to be a precursor for the development of prostate carcinoma [34,35]. Many of the genetic changes that drive prostate cancer occur early in pathogenesis and are evident in PIN cells. Interestingly, there was a more stable 2.5-fold decrease in miR-125b expression in PIN when compared to the benign epithelium. By the time of progression to tumor, the loss of miR-125b was drastically reduced (≈ a 12-fold decrease). Thus, as seen earlier, the overall level of miR-125b dropped as the oncogenic nature of the tissue increased even beginning as early as BPH and certainly by the establishment of PIN with practically complete loss by the tumor stage in this patient’s sample.

### RPPA Measurement of Protein Content in Prostate Cancer Cell Lines

Previously, gene array profiling has been used to distinguish genes that are dysregulated during the development of disease. It was traditionally thought that mRNA expression reflected actual protein levels. However, knowledge of post-transcriptional controls that affect mRNA translation challenges this traditional theory. It is now known that most genes are influenced by at least one miR. Thus, mRNA expression correlates poorly with protein levels. Direct measurement of protein content via a proteomic approach would be a better indicator of overall miR regulation than gene arrays.

To better correlate changes in miR expression with protein levels, p69 and M12 cell extracts were subjected to RPPA analysis, surveying the expression of 111 different proteins deemed important in cancer. After verification of internal standards and normalization, several proteins exhibited a significant change in expression. Of these, the most significant changes were exhibited by ErbB2, ErbB3, PI3K, p-BAD, MET, and pERK (Table 3). Although a role for the ErbB family of proteins has been well documented in breast cancer, a connection in prostate cancer has only recently been proposed [36,37].

**Table 3:** RPPA analysis shows increased protein expression from the PI3K/RAS pathways in M12 versus p69 Cell Lines.

Protein	Fold change*
ErbB2	1.8
ErbB3	2.0
PI3K	1.4
p-BAD	1.7
MET	1.3
pERK	4.0

*All p values for these comparisons are < 0.001

### Correlation of RPPA Analysis with miR Expression

miR expression analysis confirmed a substantial loss of miR-125b in relevant prostate cell lines and in actual tumor cells via LCM analysis. It has been proposed that the binding sites for miR-125b are contained within the 3’-UTRs of ErbB2 and ErbB3 [30]. Thus, a correlation between RPPA results and miR-125b expression was explored.

**Fig 4:**
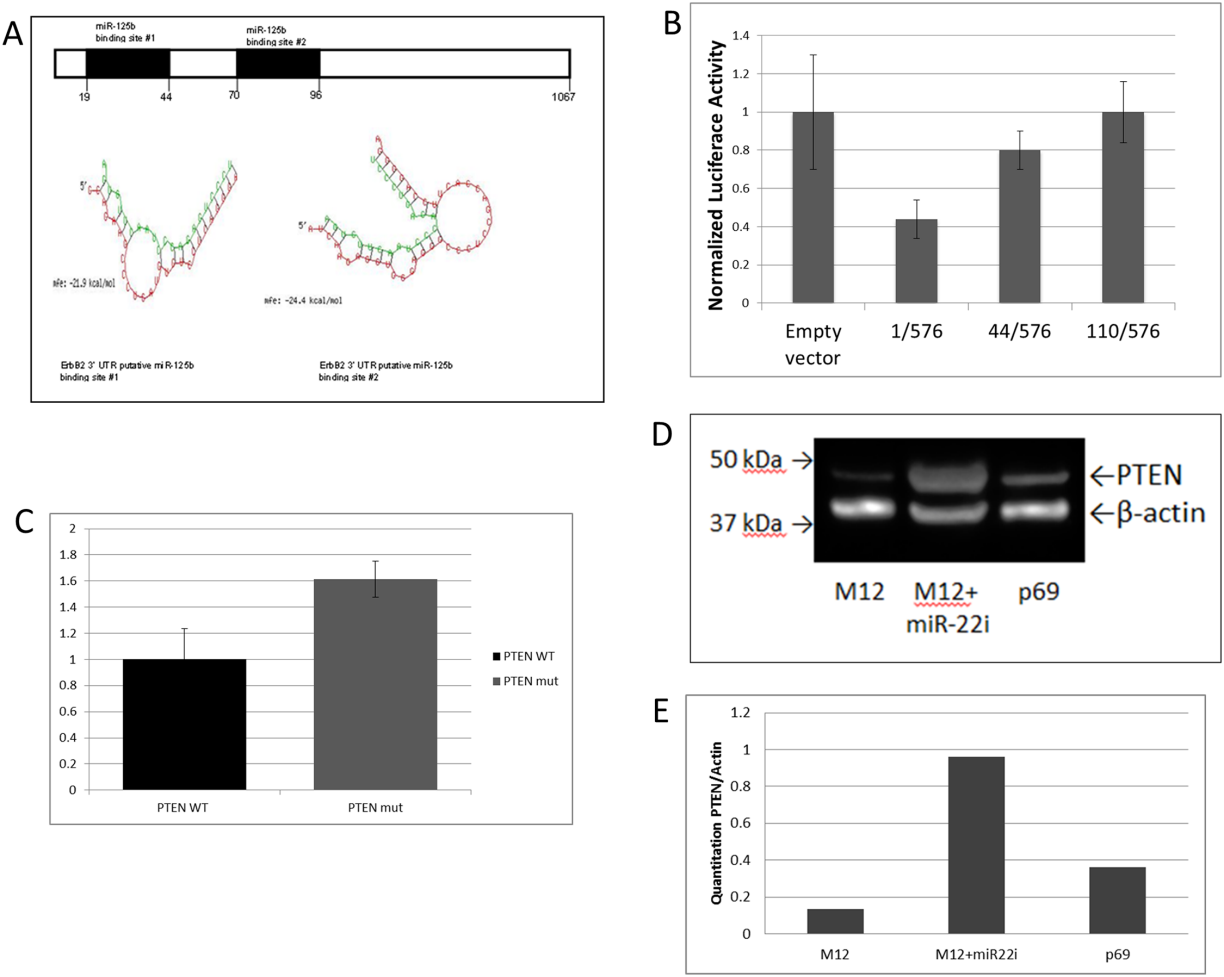
Validation of miR-125b and miR-22 binding to the 3'-UTR of ErbB2 and PTEN respectively. **(A)** Identification of two miR-125b binding sites within the 3’-UTR of ErbB2. Site 1 (position -19 to -44) downstream of the stop codon is proven by Scott et al [30]. Site 2 (position -70 to -96) is proposed here by an analysis with RNAhybrid [56]. Both sites display notable binding between ErbB2’s 3’-UTR (red) and miR-125b (green) with favorable MFEs. **(B)** The M12 cell line was transfected with a dual luciferase reporter construct containing various portions of ErbB2’s 3’-UTR as noted. Firefly luciferase expression is normalized to renilla luciferase activity. Results are the mean of 3 independent experiments each performed in triplicate. ANOVA test indicates a significant difference with a P-value < 0.05. **(C)** The effect of miR-22 binding on the wild type and mutant PTEN 3’-UTR target binding site was assessed on firefly versus renilla luciferase activity as described in panel B via transfection into the M12 cell line. Mutation of a single base (C to A) within the target to miR-22’s seed region [31] resulted in increased firefly luciferase activity. Results are the average of 3 experiments repeated in triplicate. **(D)** A representative western blot of whole cell extracts (30 μg) from M12, M12+miR-22i and p69 cells. Proteins were separated on a 4–12% Tris-Bis gel and incubated with a PTEN antibody. β-actin was used as a loading control. The position of migration of relevant protein size markers are noted. **(E)** Quantitation of western blot shown in panel D with PTEN levels normalized to β-actin.

Analysis of ErbB2’s 3’-UTR suggested, in addition to the known miR-125b binding site (site #1) at position 19–44 downstream of the stop codon, a second binding site (site #2) at position 70 to 96 was likely (Fig 4A). Both sites exhibited a perfect match between the seed region of miR-125b and the corresponding target region of ErbB2 with a favorable Molecular Free Energy (MFE) structure of -21.9 and -24.4 kcal/mol, respectively (Fig 4A). The pmirGLO plasmid containing ErbB2’s wild type 3’UTR (1/576) resulted in a considerable reduction (≈60%) in luciferase activity compared to empty vector when transfected into the M12 cell line (Fig 4B). A similar result was detected in the breast cancer cell line SKBR3-125b [30]. Deletion of site #1 (a proven target for miR-125b) in pErbB44/576 restored considerable activity (>50%), but further deletion of the potential new site#2 (110/576) was required to completely restore luciferase activity back to vector only levels.

The RPPA analysis confirmed the role of the PI3K pathway and ErbB family in prostate cancer [38,39]. PTEN is a known cellular brake for this pathway [31]. miR-22 is known to regulate PTEN activity by binding to its 3’-UTR [31]. In the M12 cell line we confirmed that mutation of a single C to an A within PTEN’s wild type binding site resulted in a considerable restoration of luciferase activity matching that obtained in the initial report by Bar et al. (Fig 4C). Since PTEN levels were not analyzed in the original RPPA, western blots confirmed that PTEN expression was significantly reduced (2.7-fold) in M12 cells compared to parental p69 cells (Fig 4D and 4E) (entire gel included as S1 Fig).

### Restoration of miR-125b or Decrease of miR-22 Affected the Migratory and Invasive Behavior of the M12 Cancer Cell Line

To further explore the role of miR-125b and miR-22 in prostate tumorigenesis, M12 stable cell lines were constructed that either increased expression of miR-125b (M12+miR125b), or expressed a proven miR-22 inhibitor, (M12+miR-22i). An analysis of an important negative control of the M12 stable cell line expressing a scrambled RNA sequence of similar length to miR125b (M12+scrambled RNA) was included and described previously as M12+NTC [22]. In the case of miR-125b restoration, qRT-PCR confirmed a 3.5-fold increase in miR-125b levels in M2+miR-125b cells versus parental M12s or the M12+scrambled RNA control.

**Fig 5:**
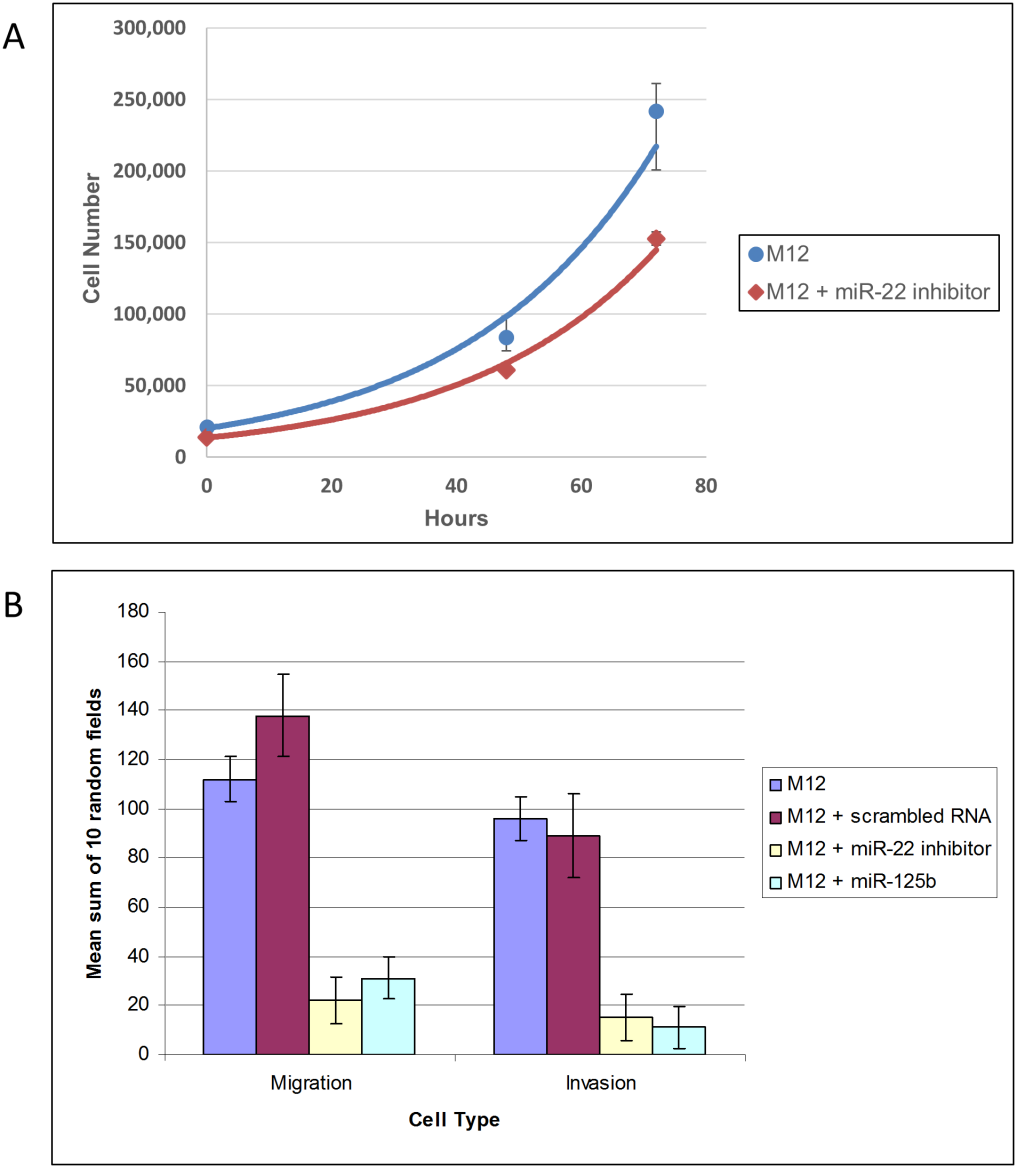
Restoration of miR-125b or inhibition of miR-22 impairs *in vitro* migratory and invasive potential of M12 cells but initially has little effect on cell proliferation. **(A)** A representative growth curve of M12 versus M12+miR-22inhibitor cells is shown. Cells (20,000) were plated in triplicate and viable cells counted at 0, 48, and 72 hours in a Beckman Coulter Vi-CELL automated cell viability analyzer using trypan blue exclusion (cell viability >94%). The average viable cell number was plotted on an exponential curve with the standard deviation noted. Analysis of cell growth at the 48 and 72 hour time points using a student t-test yielded a p-value of 0.11 and 0.009, respectively **(B)** Cells (50,000) were plated in triplicate in serum free media in the top chamber of a ThinCert^™^ tissue culture insert and processed as described in Materials and Methods. Panel A confirmed no significant difference in growth potential at 20 hours at which time the number of migrated cells was counted in 10 random fields and the total number of migratory cells estimated by summing up each of the individual fields. The average mean sum of each well is presented, along with the standard error. The invasion assay used the same experimental set up with the additional step of coating the membrane with Culturex^®^ growth factor reduced basement membrane (60 μl) prior to cell addition. Results are shown for parental M12 cells or M12 cells stably transformed with a scrambled RNA sequence as a negative control (M12+scrambled RNA), a miR-22 inhibitor (M12+miR-22 inhibitor) or restored miR-125b (M12+miR-125b). Data is the mean of 3 independent experiments, each performed in triplicate. ANOVA test indicates a significant difference with a P-value < 0.05.

The proliferation rate of M12+miR-22i versus the parental M12 cells was compared (Fig 5A). At 24 hours cell growth was equivalent yielding a p value of 0.11, but by 72 hours the proliferation rate of the modified cell line started to slow down (p value = .009) probably due to the decrease in functional miR-22 contributing to an increased level of the cellular brake PTEN (see Fig 4D and 4E). However, no differences in growth rate were detected for M12s compared to the stable M12+miR-125b cell line (negative data not shown).

Since no significant proliferative differences were noted for altering miR-125b or miR-22 expression at 20 hours, the effect of these modifications on cell migration and invasion was determined. Restoration of miR-125b resulted in a substantial decrease in migratory ability (3.7-fold) for M12+miR125b in Transwell chamber assays versus a similar decrease when the action of miR-22 is inhibited (Fig 5). Similar results were obtained for measurement of invasive ability. A scrambled RNA control either showed a slight increase (25%) in migratory behavior or no measurable effect on invasion. Western blots confirmed that upon inhibition of miR-22, PTEN activity was increased 7-fold (Fig 4D and 4E).

## Discussion

Previous network analyses of oncogenic cell lines suggested that miRs preferentially target protein nodes that regulate key processes implicated in cancer progression [11,12]. Our work substantiates this hypothesis by comparing miR array results with proteomics. To our knowledge this is one of the first studies aimed at directly correlating miR expression levels to protein quantity rather than transcript levels revealed by the usual gene arrays (Table 3). Since miR binding doesn’t necessarily result in the degradation of all target mRNAs, studies that attempt to correlate mRNA levels to miR arrays to deduce targets for miR binding may not be consistent. The more relevant comparison is miR array data compared to protein product analysis, as western blots and luciferase+3’-UTR assays have been instrumental in validating miR:mRNA targeting. Moreover, differences in the tumorigenic/metastatic properties of these related cell lines could NOT be explained by the acquisition of nucleotide mutations within known cancer genes (Table 2). This result strengthens the likelihood of differences in miR expression as major contributors to tumor progression.

This three pronged approach of network analysis supported by miR arrays and proteomics has suggested two miRs as important regulators of prostate tumor progression. The importance of miR-125b as a tumor suppressor and miR-22 as an oncomiR was initially suggested by qRT-PCR analysis of the p69, M2182 and M12 cell lines [18]. The appropriateness of this model system for accurately reflecting tumorigenesis was supported by LCM analysis of actual human prostate tumor samples. Moreover, luciferase+3’-UTR constructs validated a direct interaction between miR-125b binding to ErbB2 with the identification of a second binding site (Fig 4B). Confirmation of site#1 agrees with previously published results in SKBR3 cells, but our study suggests that complete repression by miR-125b requires at least two binding sites within ErbB2’s 3’-UTR. Similarly, miR-22 was shown to directly bind to PTEN (Fig 4C). Restoration of miR-125b or inhibition of miR-22 expression resulted in a substantial decrease in the migratory and invasive abilities of the M12 cell line (Fig 5), which could not be accounted for by strictly modifying growth rate.

**Fig 6:**
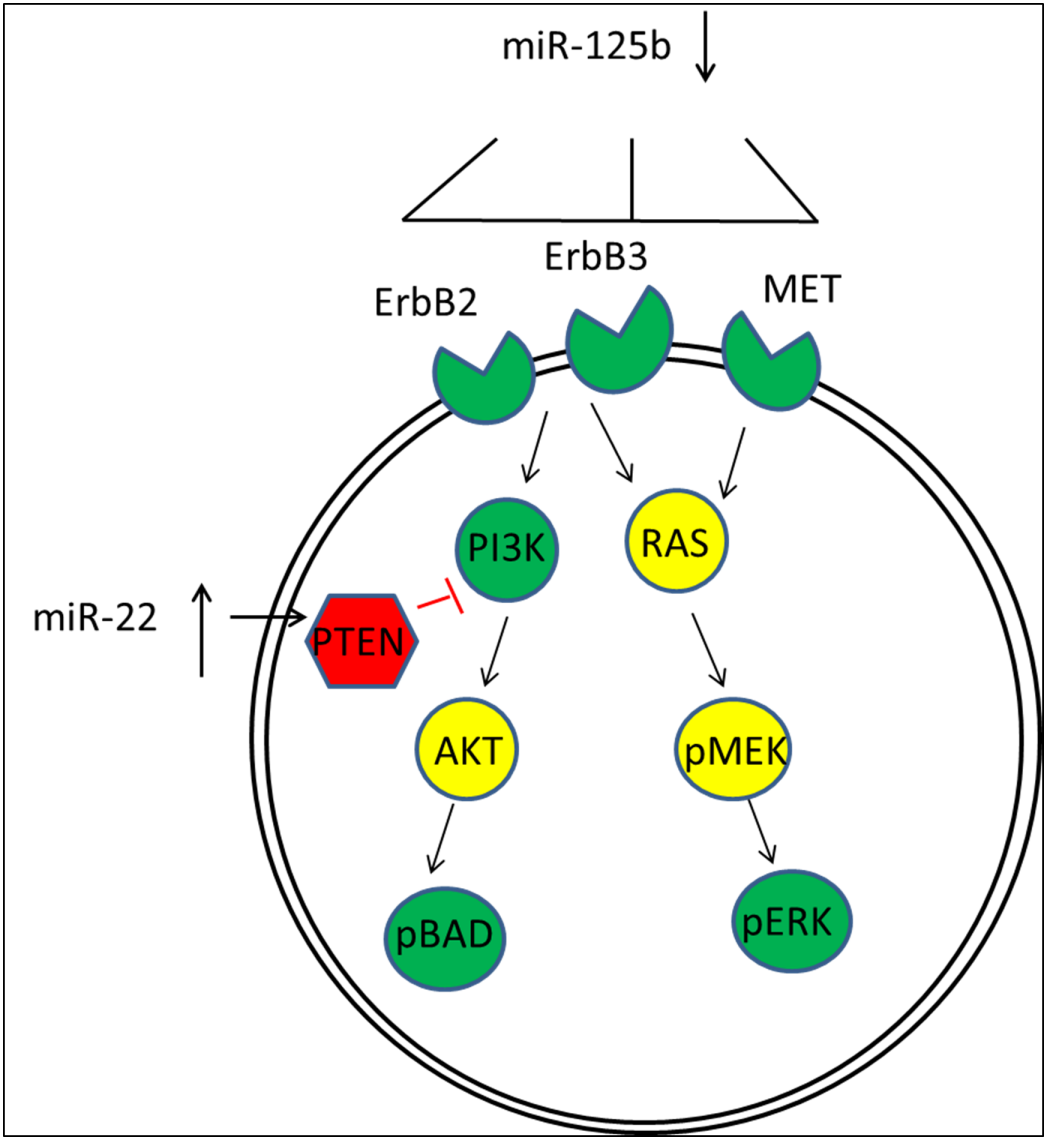
Model for dual regulation by miR-125b and miR-22. RPPA data shows increases in ErbB3, ErbB2, MET, PI3K, pBAD and pERK protein expression in M12 cells relative to the parental p69 cell lines (Table 3: green targets). Western blot (Fig 4D) confirms a decrease in PTEN expression (red). These proteins figure prominently in signal transduction pathways that are targeted by miR-125b and miR-22. Intermediates in yellow (AKT, RAS and pMEK) were not included in the proteome analysis. miR-125b directly targets ErbB2, ErbB3 and MET and miR-22 targets PTEN [30,31]. We propose that the loss of tumor suppressor miR-125b in the M12 cell line would result in enhanced signaling via the PI3K/AKT pathway and the RAS/pMEK pathway as regulated by MET. Conversely, miR-22 targets PTEN, the loss of which by increased expression of miR-22 further exacerbates PI3K signaling. In this scenario the dysregulation of these two miRs could contribute to the highly tumorigenic/metastatic phenotype displayed by the M12 cells compared to the parental p69 cell line and subsequently contribute to prostate tumorigenesis.

Altogether these results suggest an interesting cooperation between miR-125b and miR-22 in regulating key signal transduction pathways known to be dysregulated in prostate cancer progression (Fig 6). In this case, a decrease in miR-125b would permit increased expression of ErbB2/3 resulting in enhanced levels of PI3K and BAD as one signal transduction pathway increased in our proteomic data (Table 3) and in prostate tumorigenesis [39]. Concurrently, the overexpression of miR-125a/b was found to reduce ErbB2/3 at both the transcript and protein level in the human breast cancer cell line SKBR3 thereby reducing AKT signaling [30]. In addition, a noted increase in the amount of MET protein, another known target of miR-125b expression, would lead to an increase in RAS signaling thru the pMEK/pERK pathway, also shown to be increased in prostate cancer and supported by our proteomic data (Table 3). Conversely, the overexpression of miR-22 would lower PTEN levels, the cellular brake known to fine tune the PI3K/AKT pathway [40]. Reduced expression of PTEN would further result in enhancement of the PI3K signaling pathway [40]. In HEK293T cells, miR-22 gene expression has been shown to be transcriptionally upregulated by the AKT pathway in a feed forward loop, which would further decrease PTEN levels [31]. Thus, the dual dysregulation of these two miRs could easily disrupt the normal homeostasis of cellular expression contributing to an enhanced tumorigenic/metastatic phenotype. Although the importance of the PI3K/AKT and MEK/ERK pathways as known modulators of prostate cancer progression is documented [38], how the expression of these pathways could be modulated in prostate cancer has not been established. Our studies suggest a mechanism by which these signal transduction pathways are enhanced in prostate tumorigenesis due to the loss of miR-125b and overexpression of miR-22.

miR-125b has been reported to be dysregulated in a variety of cancers, either acting as an oncomiR being up-regulated in diffuse-type gastric cancer [41], colon [42], pancreatic [43], and urothelial carcinomas [44] or as a tumor suppressor being down regulated in drug-resistant ovarian cancer [45], breast [46], head and neck squamous cell carcinoma [47] and in liver cancer [48]. In breast [46] and ovarian cancer [49], promoter methylation has been shown to account for decreased miR-125b expression.

Conflicting results for miR-125b expression have been reported for prostate cancer cell lines. Shi et al found miR-125b to be increased in androgen stimulated prostate cell lines such as LNCaP or in the PC3 prostate cell line where increased miR-125b expression promoted the growth of prostate cancer xenografts by targeting pro-apoptotic genes [50,51]. Conversely, miR-125b was reported to be downregulated in prostate carcinomas comparing these same cell lines, xenografts thereof and in clinical prostate tissue samples [52], and in miR array screens of prostate tumor tissue matched to normal tissue as confirmed by qRT-PCR assays [53,54]. It is not clear why the discrepancy in results with these prostate cell lines, but it was suggested this may be affected by androgen withdrawal leading to an increase in p53 expression [51].

Due to the heterogeneity of whole prostate tumors, it is likely that differences in miR-125b expression patterns among individual whole tumors are not surprising [19,26–29,55,56]. When LCM retrieval of separate tumor from normal tissue is employed, miR-125b shows a definite decrease early in tumorigenesis, which supports miR-125b’s putative role as a tumor suppressor. Considerable miR-125b expression in stroma tissue (Fig 3B) could contribute to the variable results obtained from whole tumor analysis dependent upon the amount of contamination from stroma and other non-tumor cell types. We characteristically detected patches of tumor of different grades G3, G4, or G5 (Fig 2) [19], BPH or PIN intermixed in the same tumor sample (Fig 3B) as well as patches of lymphocytes or prostatic urethra. There is little doubt that solid tumors are heterogeneous further contributing to conflicting results. Therefore, more targeted methods like LCM, which can retrieve single cell-types, leads to improved analysis. In support of our findings, a similar LCM study found miR-125 to be down-regulated 2.2-fold in pooled FFPE prostate cancer samples compared to their normal counterparts (5 Caucasians and 5 African) [35]. However, since these results were reported for only a pooled sample, it is impossible to monitor individual sample variability or compare expression across various stages of prostate cancer as reported here, i.e., BPH versus PIN. Additional LCM studies are required to further address this hypothesis.

## Conclusion

In conclusion, our approach has uncovered the loss of miR-125b acting as a tumor suppressor and the overexpression of the oncomiR miR-22 in promoting prostate tumor progression. As more studies strive to undercover multiple targets for individual miRs, an additional level of complexity may come from the combinatorial effect of miRs, which together exert a greater effect on cancer progression than any single miR acting alone. Given that each miR is proposed to target hundreds of mRNAs, a combination of miRs would certainly have an even greater effect than any single miR. In this regard, it will be interesting to note what other miRs act in concert to further regulate tumorigenesis and metastasis.

## Supporting Information

S1 Fig Entire western blot of whole cell extracts (30 μg) from M12, M12+miR-22i and p69 cells as shown in Fig 4D. A blank and duplicate p69 lane shown here were deleted from Fig 4D.
(TIF)

## References

[1] Kent OA, Mendell JT. A small piece in the cancer puzzle: microRNAs as tumor suppressors and oncogenes. Oncogene 2006;25: 6188–6196. 17028598 10.1038/sj.onc.1209913

[2] Bartel DP, Chen CZ. Micromanagers of gene expression: the potentially widespread influence of metazoan microRNAs. Nat Rev Genet. 2004;5: 396–400. 15143321 10.1038/nrg1328

[3] Boehm M, Slack FJ. MicroRNA control of lifespan and metabolism. 2006;5: 837–840. 10.4161/cc.5.8.2688 16627994

[4] Zhao Y, Srivastava D. A developmental view of microRNA function. Trends Biochem Sci. 2007;32: 189–197. 17350266 10.1016/j.tibs.2007.02.006

[5] Esquela-Kerscher A, Slack FJ. Oncomirs—microRNAs with a role in cancer. Nat Rev Cancer. 2006;6: 259–269. 16557279 10.1038/nrc1840

[6] Bartel DP. MicroRNAs: genomics, biogenesis, mechanism, and function. Cell. 2004;116: 281–297. 14744438 10.1016/s0092-8674(04)00045-5

[7] Volinia S, Calin GA, Liu CG, Ambs S, Cimmino A, Petrocca F, et al A microRNA expression signature of human solid tumors defines cancer gene targets. Proc Natl Acad Sci U S A. 2006;103: 2257–2261. 16461460 10.1073/pnas.0510565103 PMC1413718

[8] DeVere White RW, Vinall RL, Tepper CG, Shi XB. MicroRNAs and their potential for translation in prostate cancer. Urol Oncol. 2009;27: 307–311. 19414119 10.1016/j.urolonc.2009.01.004 PMC2761743

[9] Calin GA, Sevignani C, Dumitru CD, Hyslop T, Noch E, Yendamuri S, et al Human microRNA genes are frequently located at fragile sites and genomic regions involved in cancers. Proc Natl Acad Sci U S A. 2004;101: 2999–3004. 14973191 10.1073/pnas.0307323101 PMC365734

[10] Hammond SM. MicroRNAs as oncogenes. Curr Opin Genet Dev. 2006;16: 4–9. 16361094 10.1016/j.gde.2005.12.005

[11] Budd WT, Weaver DE, Anderson J, Zehner ZE. MicroRNA dysregulation in prostate cancer: Network analysis reveals preferential regulation of highly connected nodes. Chem Biodivers. 2012;9: 857–867. 10.1002/cbdv.201100386 22589088 PMC3386794

[12] Budd WT, Seashols S, Weaver D, Joseph C, Zehner ZE. A networks method for ranking microRNA dysregulation in cancer. BMC Syst Biol. 2013;7 Suppl 5: S3-0509-0509-7-S5-S3. Epub 2013 Dec 9. 10.1186/1752-0509-7-S5-S3 PMC4028974 24564923

[13] Bae VL, Jackson-Cook CK, Brothman AR, Maygarden SJ, Ware JL. Tumorigenicity of SV40 T antigen immortalized human prostate epithelial cells: association with decreased epidermal growth factor receptor (EGFR) expression. Int J Cancer. 1994;58: 721–729. 8077059 10.1002/ijc.2910580517

[14] Bae VL, Jackson-Cook CK, Maygarden SJ, Plymate SR, Chen J, Ware JL. Metastatic sublines of an SV40 large T antigen immortalized human prostate epithelial cell line. Prostate. 1998;34: 275–282. 9496902 10.1002/(sici)1097-0045(19980301)34:4<275::aid-pros5>3.0.co;2-g

[15] Astbury C, Jackson-Cook CK, Culp SH, Paisley TE, Ware JL. Suppression of tumorigenicity in the human prostate cancer cell line M12 via microcell-mediated restoration of chromosome 19. Genes Chromosomes Cancer. 2001;31: 143–155. 11319802 10.1002/gcc.1128

[16] Paweletz CP, Charboneau L, Bichsel VE, Simone NL, Chen T, Gillespie JW, et al Reverse phase protein microarrays which capture disease progression show activation of pro-survival pathways at the cancer invasion front. Oncogene. 2001;20: 1981–1989. 11360182 10.1038/sj.onc.1204265

[17] Grubb RL, Calvert VS, Wulkuhle JD, Paweletz CP, Linehan WM, Phillips JL, et al Signal pathway profiling of prostate cancer using reverse phase protein arrays. Proteomics. 2003;3: 2142–2146. 14595813 10.1002/pmic.200300598

[18] Seashols S. Variation and modulation of microRNAs in prostate cancer and biological fluids. Phd Dissertation. Virginia Commonwealth University 2013.

[19] Zhang X, Ladd A, Dragoescu E, Budd WT, Ware JL, Zehner ZE. MicroRNA-17-3p is a prostate tumor suppressor in vitro and in vivo, and is decreased in high grade prostate tumors analyzed by laser capture microdissection. Clin Exp Metastasis. 2009;26: 965–979. 10.1007/s10585-009-9287-2 19771525

[20] Espina V, Wulfkuhle JD, Calvert VS, VanMeter A, Zhou W, Coukos G, et al Laser-capture microdissection. Nat Protoc. 2006;1: 586–603. 17406286 10.1038/nprot.2006.85

[21] Budd WT, Combinatorial analysis of tumorigenic microRNAs driving prostate cancer PhD Dissertation. Virginia Commonwealth University 2012.

[22] Zhang X, Fournier M, Ware JL, Bissel MJ, Yacoub A, Zehner ZE. Inhibition of vimentin or ß1-integrin reverts morphology of prostate tumor cells grown in laminin-rich extracellular matrix gels and reduces tumor growth *in vivo* . Mol Cancer Ther. 2009;8:499–508. 10.1158/1535-7163.MCT-08-0544 19276168 PMC2703491

[23] Livak KJ, Schmittgen TD. Analysis of relative gene expression data using real-time quantitative PCR and the 2^-(ΔΔC(T))^ Method. Methods. 2001;25: 402–408. 11846609 10.1006/meth.2001.1262

[24] Gleason DF, Mellinger G$. Prediction of prognosis for prostatic adenocarcinoma by combined histological grading and clinical staging. J Urol. 1974;111: 58–64. 4813554 10.1016/s0022-5347(17)59889-4

[25] Paweletz CP, Liotta LA, Petricoin EF 3rd. New technologies for biomarker analysis of prostate cancer progression: Laser capture microdissection and tissue proteomics. Urology. 2001;57: 160–163. 11295617 10.1016/s0090-4295(00)00964-x

[26] Yee JY, Limenta LM, Rogers K, Rogers SM, Tay VS, Led EJ. Ensuring good quality RNA for quantitative real-time PCR isolated from renal proximal tubular cells using laser capture microdissection. BMC Res Notes 2014;7: 62–69. 10.1186/1756-0500-7-62 24467986 PMC3905289

[27] Joseph A, Gnanapragasam VJ. Laser-capture microdissection and transcriptional profiling in archival FFPE tissue in prostate cancer. Methods Mol Biol. 2011;755: 291–300. 10.1007/978-1-61779-163-5_24 21761313

[28] Nonn L, Vaishnav A, Gallagher L, Gann PH. mRNA and micro-RNA expression analysis in laser-capture microdissected prostate biopsies: valuable tool for risk assessment and prevention trials. Exp Mol Pathol. 2010;88: 45–51. 10.1016/j.yexmp.2009.10.005 19874819 PMC2815196

[29] Shukla CJ, Pennington CJ, Riddick AC, Sethia KK, Ball RY, Edwards DR. Laser-capture microdissection in prostate cancer research: establishment and validation of a powerful tool for the assessment of tumour-stroma interactions. BJU Int. 2008;101; 765–74. 10.1111/j.1464-410X.2007.07372.x 18190638

[30] Scott GK, Goga A, Bhaumik D, Berger CE, Sullivan CS, Benz CC. Coordinate suppression of ERBB2 and ERBB3 by enforced expression of micro-RNA miR-125a or miR-125b. J Biol Chem. 2007;282: 1479–1486. 17110380 10.1074/jbc.M609383200

[31] Bar N, Dikstein R. miR-22 forms a regulatory loop in PTEN/AKT pathway and modulates signaling kinetics. PLoS One. 2010;5: e10859 10.1371/journal.pone.0010859 20523723 PMC2877705

[32] Wu Y, Zhang X, Zehner ZE. c-Jun and the dominant-negative mutant, TAM67, induce vimentin gene expression by interacting with the activator Sp1. Oncogene. 2003;22: 8891–8901. 14654785 10.1038/sj.onc.1206898

[33] Isaacs JT, Coffey DS. Etiology and disease process of benign prostatic hyperplasia. Prostate Suppl. 1989;2: 33–50. 2482772 10.1002/pros.2990150506

[34] Klink JC, Miocinovic R, Magi Galluzzi C, Klein EA. High-grade prostatic intraepithelial neoplasia. Korean J Urol. 2012;53: 297–303. 10.4111/kju.2012.53.5.297 22670187 PMC3364467

[35] Bostwick DG, Liu L, Brawer MK, Qian J. High-grade prostatic intraepithelial neoplasia. Rev Urol. 2004;6: 171–179. 16985598 PMC1472840

[36] Holbro T, Beerli RR, Maurer F, Koziczak M, Barbas CF 3rd, Hynes NE. The ErbB2/ErbB3 heterodimer functions as an oncogenic unit: ErbB2 requires ErbB3 to drive breast tumor cell proliferation. Proc Natl Acad Sci U S A. 2003;100: 8933–8938. 12853564 10.1073/pnas.1537685100 PMC166416

[37] Lu X, Kang Y. Epidermal growth factor signaling and bone metastasis. Br J Cancer. 2010;102: 457–461. 10.1038/sj.bjc.6605490 20010942 PMC2822931

[38] Dubovenko A, Serebryiskaya T, Nikolsky Y, Nikolskaya T, Perlina A, JeBailey L, et al Reconstitution of the ERG gene expression network reveals new biomarkers and therapeutic Targets in ERG positive prostate tumors. J Cancer. 2015;6: 490–501. 10.7150/jca.8213 26000039 PMC4439933

[39] Priolo C, Pyne S, Rose J, Regan ER, Zadra G, Photopoulos C, et al AKT1 and MYC induce distinctive metabolic fingerprints in human prostate cancer. Cancer Res. 2014;74: 7198–7204. 10.1158/0008-5472.CAN-14-1490 25322691 PMC4267915

[40] Wang S, Gao J, Lei Q, Rozengurt N, Pritchard C, Jiao J, et al Prostate-specific deletion of the murine Pten tumor suppressor gene leads to metastatic prostate cancer. Cancer Cell. 2003;4: 209–221. 14522255 10.1016/s1535-6108(03)00215-0

[41] Ueda T, Volinia S, Okumura H, Shimizu M, Taccioli C, Rossi S, et al Relation between microRNA expression and progression and prognosis of gastric cancer: a microRNA expression analysis. Lancet Oncol. 2010;11: 136–146. 10.1016/S1470-2045(09)70343-2 20022810 PMC4299826

[42] Baffa R, Fassan M, Volinia S, O'Hara B, Liu CG, Palazzo JP, et al MicroRNA expression profiling of human metastatic cancers identifies cancer gene targets. J Pathol. 2009;219: 214–221. 10.1002/path.2586 19593777

[43] Bloomston M, Frankel WL, Petrocca F, Volinia S, Alder H, Hagan JP, et al MicroRNA expression patterns to differentiate pancreatic adenocarcinoma from normal pancreas and chronic pancreatitis. JAMA. 2007;297: 1901–1908. 17473300 10.1001/jama.297.17.1901

[44] Veerla S, Lindgren D, Kvist A, Frigyesi A, Staaf J, Persson H, et al MiRNA expression in urothelial carcinomas: important roles of miR-10a, miR-222, miR-125b, miR-7 and miR-452 for tumor stage and metastasis, and frequent homozygous losses of miR-31. Int J Cancer. 2009;124: 2236–2242. 10.1002/ijc.24183 19127597

[45] Sorrentino A, Liu CG, Addario A, Peschle C, Scambia G, Ferlini C. Role of microRNAs in drug-resistant ovarian cancer cells. Gynecol Oncol. 2008;111: 478–486. 10.1016/j.ygyno.2008.08.017 18823650

[46] Zhang Y, Yan LX, Wu QN, Du ZM, Chen J, Liao DZ, et al miR-125b is methylated and functions as a tumor suppressor by regulating the ETS1 proto-oncogene in human invasive breast cancer. Cancer Res. 2011;71: 3552–3562. 10.1158/0008-5472.CAN-10-2435 21444677

[47] Chen D, Cabay RJ, Jin Y, Wang A, Lu Y, Shah-Khan M, et al MicroRNA deregulations in head and neck squamous cell carcinomas. J Oral Maxillofac Res. 2013;4: e2. 10.5037/jomr.2013.4102 PMC3886106 24422025

[48] Li W, Xie L, He X, Li J, Tu K, Wei L, et al Diagnostic and prognostic implications of microRNAs in human hepatocellular carcinoma. Int J Cancer. 2008;123: 1616–1622. 10.1002/ijc.23693 18649363

[49] He J, Xu Q, Jing Y, Agani F, Qian X, Carpenter R, et al Reactive oxygen species regulate ERBB2 and ERBB3 expression via miR-199a/125b and DNA methylation. EMBO Rep. 2012;13: 1116–1122. 10.1038/embor.2012.162 23146892 PMC3512405

[50] Shi XB, Xue L, Yang J, Ma AH, Zhao J, Xu M, et al An androgen-regulated miRNA suppresses Bak1 expression and induces androgen-independent growth of prostate cancer cells. Proc Natl Acad Sci U S A. 2007;104: 19983–19988. 18056640 10.1073/pnas.0706641104 PMC2148409

[51] Shi XB, Xue L, Ma AH, Tepper CG, Kung HJ, White RW. miR-125b promotes growth of prostate cancer xenograft tumor through targeting pro-apoptotic genes. Prostate. 2011;71: 538–549. 10.1002/pros.21270 20886540 PMC3017658

[52] Porkka KP, Pfeiffer MJ, Waltering KK, Vessella RL, Tammela TLJ, Visakorpi T. MicroRNA expression profiling in prostate cancer. Cancer Res. 2007;67: 6130–6135. 17616669 10.1158/0008-5472.CAN-07-0533

[53] Schaefer A, Jung M, Mollenkopf HJ, Wagner I, Stephan C, Jentzmik F, et al Diagnostic and prognostic implications of microRNA profiling in prostate carcinoma. Int J Cancer. 2010;126: 1166–1176. 10.1002/ijc.24827 19676045

[54] Ozen M, Creighton CJ, Ozdemir M, Ittmann M. Widespread deregulation of microRNA expression in human prostate cancer. Oncogene. 2008;27: 1788–1793. 17891175 10.1038/sj.onc.1210809

[55] Srivastava A, Goldberger H, Dimtchev A, Ramalinga M, Chijioke J, Marian C, et al MicroRNA profiling in prostate cancer—the diagnostic potential of urinary miR-205 and miR-214. PLoS One. 2013;8: e76994 10.1371/journal.pone.0076994 24167554 PMC3805541

[56] Kruger J, Rehmsmeier M. RNAhybrid: microRNA target prediction easy, fast and flexible. Nucleic Acids Res. 2006;34: W451–4. 16845047 10.1093/nar/gkl243 PMC1538877

