# Supplementary figures and images for: Dual Action of miR-125b As a Tumor Suppressor and OncomiR-22 Promotes Prostate Cancer Tumorigenesis
Source: PLoS One. 2015 Nov 6;10(11):e0142373. doi: 10.1371/journal.pone.0142373 (PMC4636224; doi:10.1371/journal.pone.0142373)

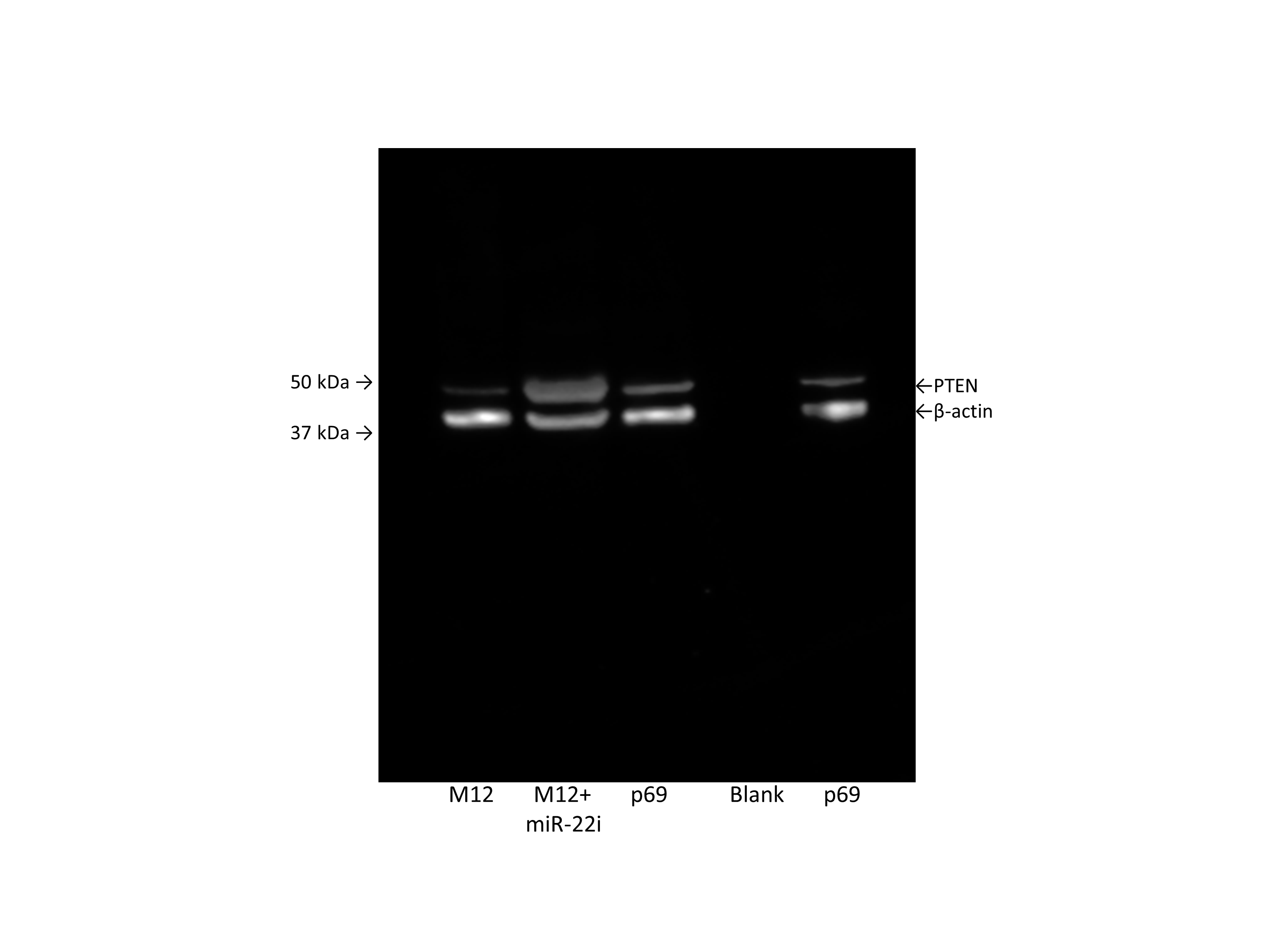

Supplement: S1 Fig — A blank and duplicate p69 lane shown here were deleted from Fig 4D. (TIF) [file pone.0142373.s001.tif]
